# Suppression of overlearning in independent component analysis used for removal of muscular artifacts from electroencephalographic records

**DOI:** 10.1371/journal.pone.0201900

**Published:** 2018-08-14

**Authors:** Jan Sebek, Radoslav Bortel, Pavel Sovka

**Affiliations:** Dept. of Circuit Theory, Czech Technical University, Faculty of Electrical Engineering, Prague, Czech Republic; University of British Columbia, CANADA

## Abstract

This paper addresses the overlearning problem in the independent component analysis (ICA) used for the removal of muscular artifacts from electroencephalographic (EEG) records. We note that for short EEG records with high number of channels the ICA fails to separate artifact-free EEG and muscular artifacts, which has been previously attributed to the phenomenon called overlearning. We address this problem by projecting an EEG record into several subspaces with a lower dimension, and perform the ICA on each subspace separately. Due to a reduced dimension of the subspaces, the overlearning is suppressed, and muscular artifacts are better separated. Once the muscular artifacts are removed, the signals in the individual subspaces are combined to provide an artifact free EEG record. We show that for short signals and high number of EEG channels our approach outperforms the currently available ICA based algorithms for muscular artifact removal. The proposed technique can efficiently suppress ICA overlearning for short signal segments of high density EEG signals.

## Introduction

During the measurement of head surface potentials with electroencephalography (EEG), weak brain signals are often corrupted by various sources of interference. One of the most common interference sources are potentials generated by muscles contracted by a measured subject. These are so called muscular or electromyographic (EMG) artifacts. The EMG artifacts are commonly occurring nuisance in many EEG records; therefore, good methods for their removal are valuable tools for EEG processing.

In the past it was suggested to use the independent component analysis (ICA) for the EMG artifact removal. Under suitable circumstances the ICA was demonstrated as a good approach for this task, and its use was developed in many works [1–11].

There are however limitations to what the ICA can achieve. If the signal length is not sufficient with respect to the number of EEG channels, the ICA suffers from so called overlearning [12–14], which prevents the separation and subsequent removal of EMG artifacts.

In the reminder of this section we introduce the problem of artifact removal with ICA more formally, specify the problem of overlearning, and review the current state-of-the-art methods in which this problem is addressed.

Let **x**[*n*] = [*x*_1_[*n*],…,*x*_*M*_[*n*]]^*T*^, *n* = 1, …, *N* denote EEG signals measured using *M* EEG electrodes. We assume that these signals are created by a linear mixture of source signals **s**[*n*] = [*s*_1_[*n*],…,*s*_*M*′_[*n*]]^*T*^

x[n]=As[n], (1)

where **A** denotes an *M*x*M*′ mixing matrix. Note that we do not expect the number of sources *M*′ to be less or equal to the number of signals *M*. This is a common assumption in the derivation of the ICA, but cannot be realistically expected in the addressed problem. We further assume that the source signals *s*_*m*_[*n*] are either generated by a brain or by other interfering sources (e.g. contracted muscles).

Our task is to remove the artifacts from the brain signals. As mentioned above, for this purpose it is possible to use the ICA, which allows to estimate a separation matrix **B** that transforms signals **x**[*n*] into source components $\hat{s}\left[n\right]={\left[{\hat{s}}_{1}\left[n\right],\ldots ,{\hat{s}}_{M}\left[n\right]\right]}^{T}$ that have their mutual dependence minimized

$$\hat{s}\left[n\right]=Bx\left[n\right].$$ (2)

If the measured signals **x**[*n*] are sufficiently long, this approach was reported to achieve a fairly good separation of brain signals and artifacts [2, 6]—this means that the most of the components ${\hat{s}}_{m}\left[n\right]$ contain either brain signals or artifacts, while the mutual intermixing of these signals is noticeably reduced. Thus, it is possible to either manually [10, 11], or automatically [11] classify each ${\hat{s}}_{m}\left[n\right]$ as either a brain signal or an artifact, and retain only the brain signals, which we will denote as $\tilde{s}\left[n\right]={\left[{\tilde{s}}_{1}\left[n\right],\ldots ,{\tilde{s}}_{M}\left[n\right]\right]}^{T}$

$${\tilde{s}}_{m}\left[n\right]=\{\begin{array}{cc} {\hat{s}}_{m}\left[n\right], & \text{if}\;{\hat{s}}_{m}\left[n\right]\;\text{is}\;\text{a}\;\text{brain}\;\text{signal},\\ 0, & \text{if}\;{\hat{s}}_{m}\left[n\right]\;\text{is}\;\text{an}\;\text{artifact}. \end{array}$$ (3)

With brain signal components $\tilde{s}\left[n\right]$ we can try to reconstruct the measured signals without the artifacts

$$\tilde{x}\left[n\right]=B^{-1}\tilde{s}\left[n\right],$$ (4)

where $\tilde{x}\left[n\right]={\left[{\tilde{x}}_{1}\left[n\right],\ldots ,{\tilde{x}}_{M}\left[n\right]\right]}^{T}$ are the estimates of the measured signals with artifacts suppressed.

The above-mentioned methodology was used before [6, 11, 15, 16] with a certain success; however, it will work only if the measured signals are sufficiently long compared to the number of measured channels. This limitation is illustrated on the following two examples.

**Fig 1:**
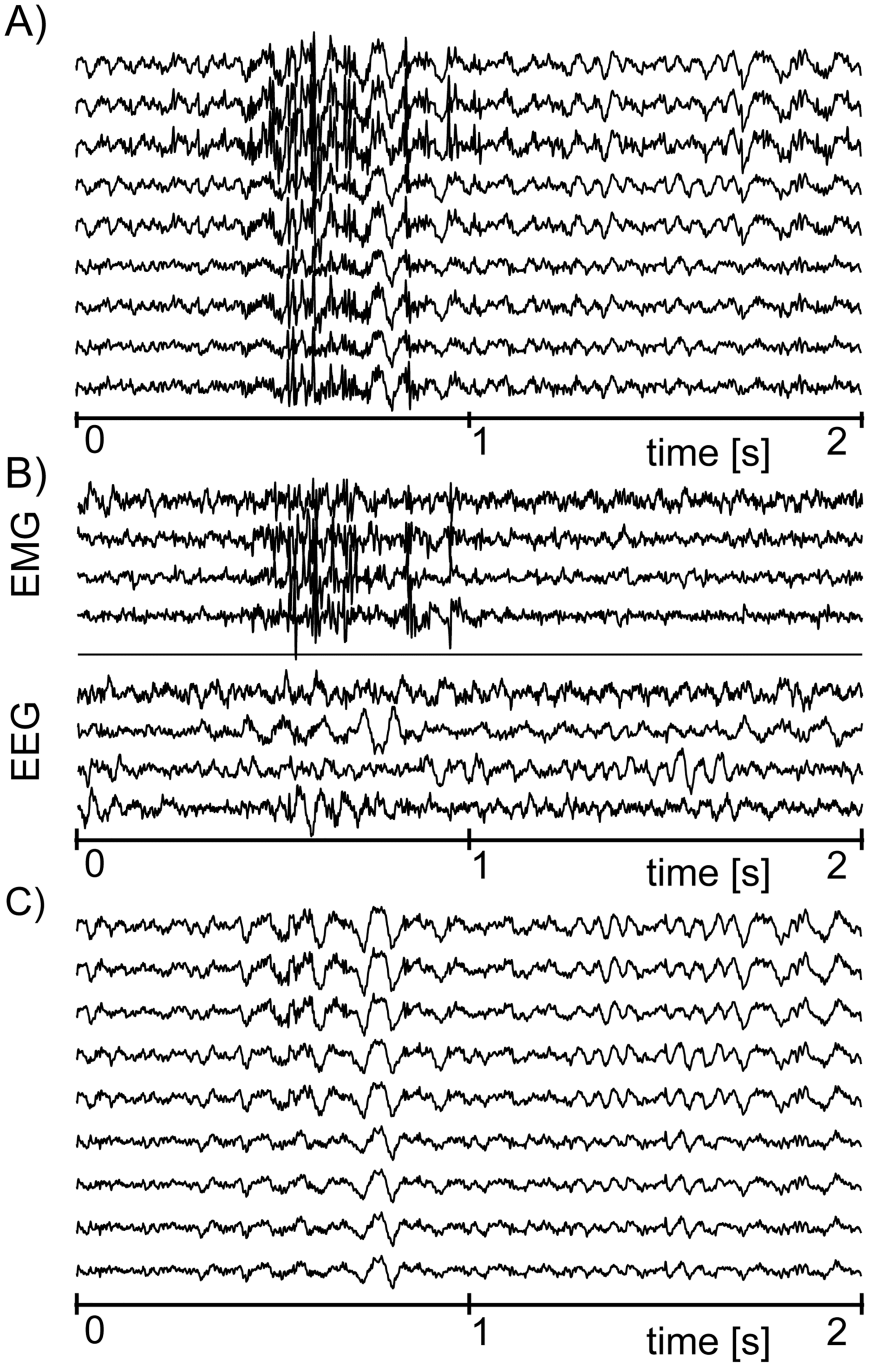
An example of muscular artifact suppression using the ICA. (A) The original EEG signals **x**[*n*] corrupted by EMG artifacts. (B) The separated source components $\hat{s}\left[n\right]$, which were classified as brain signals (denoted as EEG) and muscular artifacts (denoted as EMG). (C) The reconstructed EEG signals $\tilde{x}\left[n\right]$ with suppressed muscular artifacts.

Example 1: We have used a 2s long record from 9 EEG electrodes located in the frontal area. This record contains a clearly visible muscular artifact present in each of the 9 channels. We applied the above-mentioned method to the data (the ICA was implemented using the popular FastICA algorithm [17], Eq (14, 21, 24)]). Fig 1A shows the original signals **x**[*n*], Fig 1B shows the separated components $\hat{s}\left[n\right]$, and Fig 1C shows the reconstructed signals $\tilde{x}\left[n\right]$. In this example the muscular artifacts were suppressed, and we have obtained a nice looking reconstruction of the original EEG.

**Fig 2:**
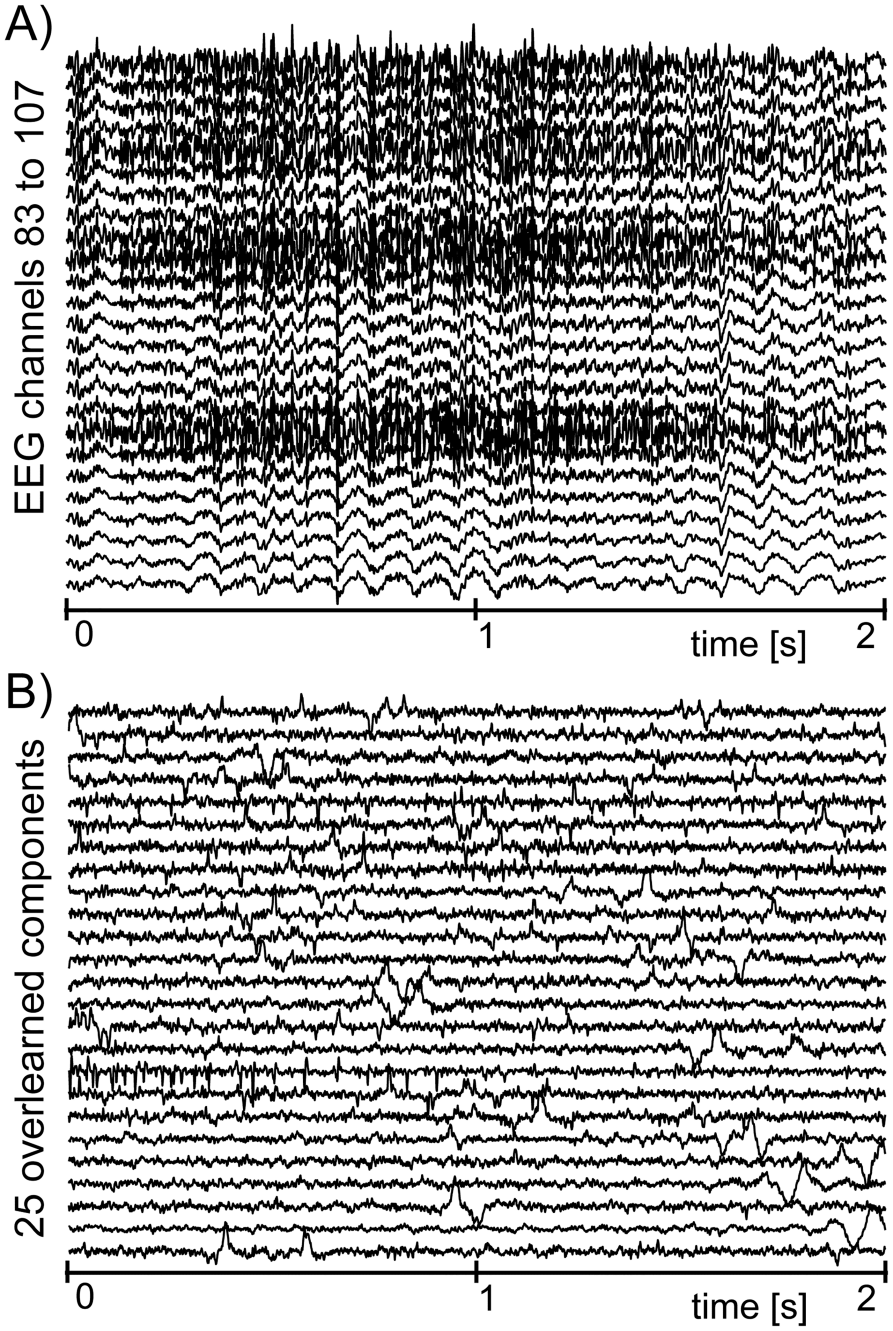
An example of a failed attempt to separate muscular artifacts from 111 channel EEG record. (A) EEG signals **x**[*n*] corrupted by EMG artifacts (for spatial reasons only 25 out of 111 EEG channels are shown). (B) The source components $\hat{s}\left[n\right]$ provided by ICA (again for spatial reasons only 25 out of 111 components are shown; the remaining components look very similar to those that are presented—they are composed of occasional bumps, and no clear separation of EEG and muscular artifacts can be recognized).

Example 2: We have used another 2s long record, but this time we used a 111 electrode array covering the frontal, parietal, temporal and occipital regions of subject’s head. As in the previous example, the record contains a clearly visible muscular artifact. We again applied the above-mentioned methodology; however, the outcome was less successful. The original signals **x**[*n*] (shown in Fig 2A) were separated into source signals $\hat{s}\left[n\right]$ (Fig 2B); however, this time we obtained no clear separation between EEG and EMG. In fact, the resulting waveforms resemble neither EEG nor EMG, and are merely composed of occasionally repeated ‘bumps’.

What we illustrated in Example 2 is a phenomenon known as overlearning [12–14]. The overlearning occurs when the ICA algorithm has too many degrees of freedom, which allows it to find components $\hat{s}\left[n\right]$ that appear to be even more independent than if $\hat{s}\left[n\right]$ contained separated EEG and EMG signals.

There are several ways how to tackle the problem of overlearning.

First, we could use longer EEG records. This, however, is not desirable. While the above-mentioned algorithm might sometimes be able to suppress muscular artifacts, it will inevitably distort the reconstructed EEG. For example, by removing 4 of the 9 components ${\hat{s}}_{m}\left[n\right]$ in the Example 1 we have reduced the rank of the reconstructed data $\tilde{x}\left[n\right]$ from the original 9 to 5 (note that the rank of an *M* dimensional signal **x**[*n*] with length *N* we understand the rank of the matrix [*x*_*m*_[*n*]]_*m*=1…*M*,*n*=1…*N*_). Thus even though the result may be ‘nice looking’ some loss of information in the reconstructed signals must have occurred. Therefore, we would like to limit the application of muscular artifact removal and the corresponding rank reduction only to the time intervals, where the muscular artifacts have really occurred, avoiding unnecessary distortion of the artifact free EEG. Better yet, the interval length should not exceed the time during which the EMG artifact origin position (and the corresponding mixing matrix **A**) is close to stationary. These time intervals can be fairly short, often less than a few seconds [18].

Next, in the past there were several published works that suggested how to suppress overlearning.

Works [12–14, 19] suggested that the proper choice of contrast functions in the ICA algorithm can decrease the level of overlearning. However, the improvement provided is often not sufficient to completely solve the problem. In our Example 2 we have used the FastICA algorithm with robust approximation of negentropy as the contrast function (which was suggested as the best choice in [12]); however, the overlearning is still distinctly present.

Works [13, 14] suggested to use the PCA to decrease the dimensionality of **x**[*n*] prior to the ICA computation. When the dimension reduction is sufficient, this approach will undoubtedly suppress overlearning; however, this will be at the cost of EEG distortion. The brain signals may have smaller power than the EMG artifacts, thus the dimensionality reduction will inevitably remove some energy from EEG, possibly removing useful information that may be missing in a subsequent EEG analysis. Thus, the application of the PCA for the dimension reduction is not always desirable, especially, if there is a way to avoid it.

Works [13, 14] also noted that the ‘bumps’ observable in the separated components produced by an overlearned ICA may have their energy predominantly at low frequencies, and therefore suggested to high-pass filter the analyzed signals prior to the ICA application. It was suggested to use either a 1Hz fixed cut-off frequency high-pass filter or an AR-process based high pass filter. It was further claimed that this high-pass filtering lessens the overlearning. In our experience this approach is not sufficient. In Example 2, the signals were already high pass filtered with a 1Hz cut-off filter (to suppress the baseline wonder), but the overlearning has still occurred. The ‘bumps’ in the separated components are simply faster (with duration of about 50ms); therefore, their occurrence cannot be prevented by prior high pass filtering without damaging useful brain signals.

Work [3], which concentrated on the suppression of ocular artifacts, suggested to use a PCA based dimension reduction to extract 3 principal components with the highest power, separate these components using the ICA, and then subtract the separated components from the original signals using a linear regression. This method is peculiar in that it uses no artifact classification. It assumes that the ocular artifacts will have the highest power, and thus will dominate the strongest principal components. Due to a distinct dimension reduction by the PCA, this methodology was claimed to be resilient to overlearning; however, we do not find it useful for other than ocular artifacts—not all EMG (or other) artifacts have power higher than brain signals, and consequently they may not get included into a few strongest principal components. In fact, with weaker artifacts, the strongest principal components may contain mostly brain signals, the removal of which would lead to the corruption of artifact free EEG signals. Still, for the sake of completeness, we include this method in our evaluation of the current state-of-the-art.

Last, there are some methods that try to alter the basic approach (2)–(4). The wavelet enhanced ICA [7, 8] extends the brain signal selection by adding wavelet filtering. Surface Laplacian [10] was also proposed to filter the EEG channels after the basic approach (2)–(4) was applied. These techniques, however, have no effect on the overlearning, which already occurs in the separation step (2). Another technique, the ensemble empirical mode decomposition and subsequent ICA [20], first decomposes each of the measured signals **x**[*n*] into the intrinsic mode functions, and then the ICA is applied. This does affect overlearning, but in a negative way, because the number of signals separated by ICA increases, which makes the effects of overlearning more severe.

All in all, in our opinion, none of the above-mentioned methods provides an efficient way to suppress the ICA overlearning without problematic side effects. We have therefore developed our own approach to the ICA overlearning suppression, which seems to outperform the above-mentioned methods, and provides better EEG reconstruction of short EEG records with EMG artifacts.

## Methods

In this section we will first describe the suggested algorithm, then point out some of its properties, and suggest how to choose its parameters.

### Suggested algorithm

We propose to separate the *M*-dimensional signal **x**[*n*] into *K* subspaces with dimension *L*

$$X_{k}\left[n\right]=P_{k}x\left[n\right],\;k=1,\ldots ,K,$$ (5)

where **P**_*k*_ are *L*x*M* subspace projection matrices (we assume that their rank is *L*, and their choice will be discussed in subsection Choice of subspaces), and $X_{k}\left[n\right]$ are *L* dimensional signals.

Next, we apply the ICA separately to each signal $X_{k}\left[n\right]$

$${\;\hat{\;S}}_{k}\left[n\right]=B_{k}X_{k}\left[n\right],$$ (6)

where **B**_*k*_ is a separation matrix estimated for $X_{k}\left[n\right]$ (note that this means that the ICA will be computed *K* times for each $X_{k}\left[n\right]$ separately).

After, we classify ${\;\hat{\;S}}_{k}\left[n\right]={\left[{\hat{s}}_{k,1}\left[n\right],\ldots ,{\hat{s}}_{k,L}\left[n\right]\right]}^{T}$ as either brain signals or artifacts, and retain only brain signals ${\;\tilde{\;S}}_{k}\left[n\right]={\left[{\tilde{s}}_{k,1}\left[n\right],\ldots ,{\tilde{s}}_{k,L}\left[n\right]\right]}^{T}$

$${\tilde{s}}_{k,i}\left[n\right]=\{\begin{array}{cc} {\hat{s}}_{k,i}\left[n\right] & \text{if}\;{\hat{s}}_{k,i}\left[n\right]\;\text{is}\;\text{a}\;\text{brain}\;\text{signal},\\ 0 & \text{if}\;{\hat{s}}_{k,i}\left[n\right]\;\text{is}\;\text{an}\;\text{artifact}. \end{array}$$ (7)

This operation can be expressed in a matrix form as

$${\;\tilde{\;S}}_{k}\left[n\right]=Q_{k}{\;\hat{\;S}}_{k}\left[n\right],$$ (8)

where **Q**_*k*_ is a diagonal matrix with its diagonal comprised of zeros and ones, positioned so that the signals ${\hat{s}}_{k,i}\left[n\right]$ classified as artifacts are eliminated according to (7).

Now, we reconstruct signals ${\;\tilde{\;X}}_{k}\left[n\right]$, in which the muscular artifacts are suppressed

$${\;\tilde{\;X}}_{k}\left[n\right]=B_{k}^{-1}{\;\tilde{\;S}}_{k}\left[n\right].$$ (9)

The above-mentioned sequence of steps can be expressed as

$${\;\tilde{\;X}}_{k}\left[n\right]=B_{k}^{-1}Q_{k}B_{k}P_{k}x\left[n\right]=C_{k}P_{k}x\left[n\right],$$ (10)

where we denoted $C_{k}=B_{k}^{-1}Q_{k}B_{k}$.

Last, we combine the individual signals ${\;\tilde{\;X}}_{k}\left[n\right]$, reconstructing the artifact free EEG. For this purpose we denote

$$\begin{array}{c} P=[\begin{array}{c} P_{1}\\ P_{2}\\ \vdots \\ P_{K} \end{array}],\;C=[\begin{array}{cccc} C_{1} & 0 & \ldots & 0\\ 0 & C_{2} & \ldots & 0\\ \vdots & \vdots & \ddots & \vdots \\ 0 & 0 & \ldots & C_{K} \end{array}], \end{array}$$ (11)

$$\;\tilde{\;X}\left[n\right]=[\begin{array}{c} {\;\tilde{\;X}}_{1}\left[n\right]\\ {\;\tilde{\;X}}_{2}\left[n\right]\\ \vdots \\ {\;\tilde{\;X}}_{K}\left[n\right] \end{array}].$$ (12)

Using notations (11) and (12), all the above-mentioned steps can be expressed as

$$\;\tilde{\;X}\left[n\right]=CPx\left[n\right].$$ (13)

To combine the elements of $\;\tilde{\;X}\left[n\right]$ into the reconstructed signals $\tilde{x}\left[n\right]$, we reverse the projection into subspaces (matrix P) by employing the Moore-Penrose pseudoinverse of P

$$\tilde{x}\left[n\right]=P^{†}\;\tilde{\;X}\left[n\right],\;\text{where}\;P^{†}={\left(P^{T}P\right)}^{-1}P^{T}.$$ (14)

Thus, the entire transformation that suppresses the artifacts can be expressed as

$$\tilde{x}\left[n\right]=P^{†}CPx\left[n\right]=Dx\left[n\right],$$ (15)

where we denoted $D=P^{†}CP$.

### Notes about properties of suggested algorithm

Since the dimension *L* of the subspaces is smaller than the dimension *M* of the original signals **x**[*n*], the ICA that is used to find the separation matrices **B**_*k*_ may be less prone to overlearning. In essence by choosing *L* sufficiently small, the problems that we illustrated in our Example 2 disappear, and the ICA behaves as in low dimensional case shown in Example 1.

If the subspace projection matrices **P**_*k*_ are chosen so that P has full rank, and if no artifacts are found in the separated signals ${\;\hat{\;S}}_{k}\left[n\right]$, then ${\;\tilde{\;S}}_{k}\left[n\right]={\;\hat{\;S}}_{k}\left[n\right]$ (i.e. **Q**_*k*_ are identity matrices), **C**_*k*_ and C become identity matrices, and D becomes the identity matrix. Consequently, no distortion is introduced into the processed signals. This is a great advantage over the PCA based overlearning suppression approach, where the rank of measured signals is always reduced, irrespective to the number of components that are being removed (i.e. the rank is reduced even if no artifacts are being removed), which causes unavoidable distortion of the resulting reconstructed signals.

If artifacts are detected in the source signal ${\;\hat{\;S}}_{k}\left[n\right]$ the rank of ${\;\tilde{\;S}}_{k}\left[n\right]$ and C will be reduced. The rank of D may then also be reduced; however, in the Results section we will show that the newly proposed method causes dimensionality reduction much less severe than the one caused by the PCA based overlearning suppression.

### Choice of subspaces

Three points should be observed when choosing the subspaces defined by the projection matrix P.

1. The dimension *L* should be small enough so that the overlearning does not pose a problem. It should however remain big enough so that there is still a sufficient number of signals so that the ICA can separate brain signals and artifacts.
2. The matrix P should be full rank (otherwise D would not be identity when no artifacts are being removed, and unnecessary EEG distortion would occur).
3. The orientation of subspaces should be chosen so that the individual ICAs can achieve good separation between brain signals and artifacts in separated signals ${\;\hat{\;S}}_{k}\left[n\right]$ even with the reduced dimension *L* of $X_{k}\left[n\right]$.

To address point (i), in this paper we identify the optimal range of values *L* by applying the algorithm to EEG data, and evaluating for which *L* we obtain the best reconstruction of brain signals. In the following sections we will introduce a methodology for the evaluation of the quality of brain signal reconstruction, and we will show that even for various EEG electrode systems we can identify a fixed range of values of *L* where the algorithm always performs well. The reader can then use the value of *L* suggested by our evaluation, and the optimization of *L* does not need to be repeated.

Point (ii) can be achieved by construction of P—simply by choosing sufficient number of sufficiently diverse subspaces, P can be full rank.

Point (iii) is somewhat more difficult to tackle. In this paper we would like to take a simple approach that is based on our experience with processing of EEG data, and suggest a choice that can be shown to produce good quality of reconstruction of brain signals. Specifically, we have observed that when we use a high density EEG array (e.g. with 111 electrodes), but limit ourselves to a small group of adjacent electrodes (e.g. 10-15 adjacent electrodes), the ICA usually provides a nice separation of brain signals and muscular artifacts. We have actually already illustrated this situation in our Example 1 which shows signals from a subgroup of 9 adjacent electrodes chosen from the 111 electrode array used in Example 2. In this paper we would therefore suggest the following steps to choose the matrices **P**_*k*_.

First, let us denote *d*_*ij*_ the Euclidean distance between *i*-th and *j*-th electrode. Next, we define vectors

$$\ell_{k}=\left[\ell_{k,1},\ldots ,\ell_{k,L}\right],$$ (16)

where *ℓ*_*k*,*i*_, *i* = 1,…, *L* are indexes of *L* electrodes with the smallest distance $d_{\ell_{k,i,}k}$ from the *k*-th electrode (with *k*-th electrode included). Now, we define the vectors

$$p_{k,i}=\left[p_{k,i,1},\ldots ,p_{k,i,M}\right],\;p_{k,i,j}=\{\begin{array}{cc} 1, & \text{if}\;\ell_{k,i}=j,\\ 0, & \text{otherwise}, \end{array}$$ (17)

and construct the projection matrices as

$$P_{k}=[\begin{array}{c} p_{k,1}\\ \vdots \\ p_{k,L} \end{array}],\;k=1,\ldots ,M.$$ (18)

Thus, each projection matrix **P**_*k*_ will extract a subspace composed of *L* signals from a group of *L* electrodes closest to the *k*-th electrode (*k*-th electrode included).

Before continuing we will make a few notes about the projection matrices **P**_*k*_ given by (18).

Note that each **p**_*k*,*i*_ (i.e. each row of **P**_*k*_) is composed of zeros and a single value of 1. Thus, the columns of **P**_*k*_ are orthogonal, and consequently the columns of P are orthogonal as well. Further, because *k* = 1,…, *M*, each column of P contains at least a single value of 1, and so has a nonzero norm. Consequently, P is a full rank matrix, just as we have required in point (ii).

We do not claim that our choice of **P**_*k*_ is optimal, but we will illustrate and claim that it provides better results than all the above-mentioned state-of-the-art methods available today. Further optimization of **P**_*k*_ may be possible, but it will not be addressed in this paper.

Last, some additional rationale for this choice of subspaces can be provided. The energy of surface EEG is typically dominated by shallow brain sources and artifacts. The head surface regions affected by these sources are typically somewhat spread due to the volume conduction; however, the greatest surface potential changes are still somewhat localized to the proximity of the origin of the source [21]. By choosing each subspace as the signals from a group of adjacent electrodes, we try to minimize the number of sources that dominate the signals in a subspace (at least as opposed to a subspace that would be composed of signals from nonadjacent electrodes spread all around the subject’s head). Consequently, it becomes easier for the ICA to separate brain signals and artifacts as was requested in point (iii).

### ICA algorithm

To find the separation matrices **B**_*k*_, we suggest to use the FastICA [17, Eq (14, 21, 24)] on data that were pre-whitened by PCA [22]. This is a popular approach, with fast convergence and previous successes in artifact separation [2, 3]. Note that the suggested methodology may work with some other ICA algorithm, but testing the performance with other ICA algorithms is out of the scope of this paper. It is also noteworthy that works [12–14] reported that the overlearning does not seem to be dependent on the choice of a specific ICA algorithm.

### Artifact classification

To decide whether the separated components ${\;\hat{\;S}}_{k}\left[n\right]$ are of a brain or a muscular origin, we used a simple classifier that compares the power of a signal at lower and higher frequencies. Compared to clean EEG, the EMG artifacts are known to have higher energy at higher frequency bands [11, 18, 23], which allows their easy identification.

We first filtered each signal ${\hat{s}}_{k,i}\left[n\right]$ into three frequency bands 3-30Hz, 60-90Hz and 110-140Hz. Then, we estimated the average power of the signals in each of these bands as *P*_*k*,*i*,1_, *P*_*k*,*i*,2_ and *P*_*k*,*i*,3_, respectively. Next, we computed the minimum of power ratios

$$\alpha_{k,i}=\min\left(P_{k,i,1}/P_{k,i,2},P_{k,i,1}/P_{k,i,3}\right).$$ (19)

Last, the values of *α*_*k*,*i*_ were compared with a chosen threshold *T* to determine whether ${\hat{s}}_{k,i}\left[n\right]$ is of a brain or a muscular origin.

**Fig 3:**
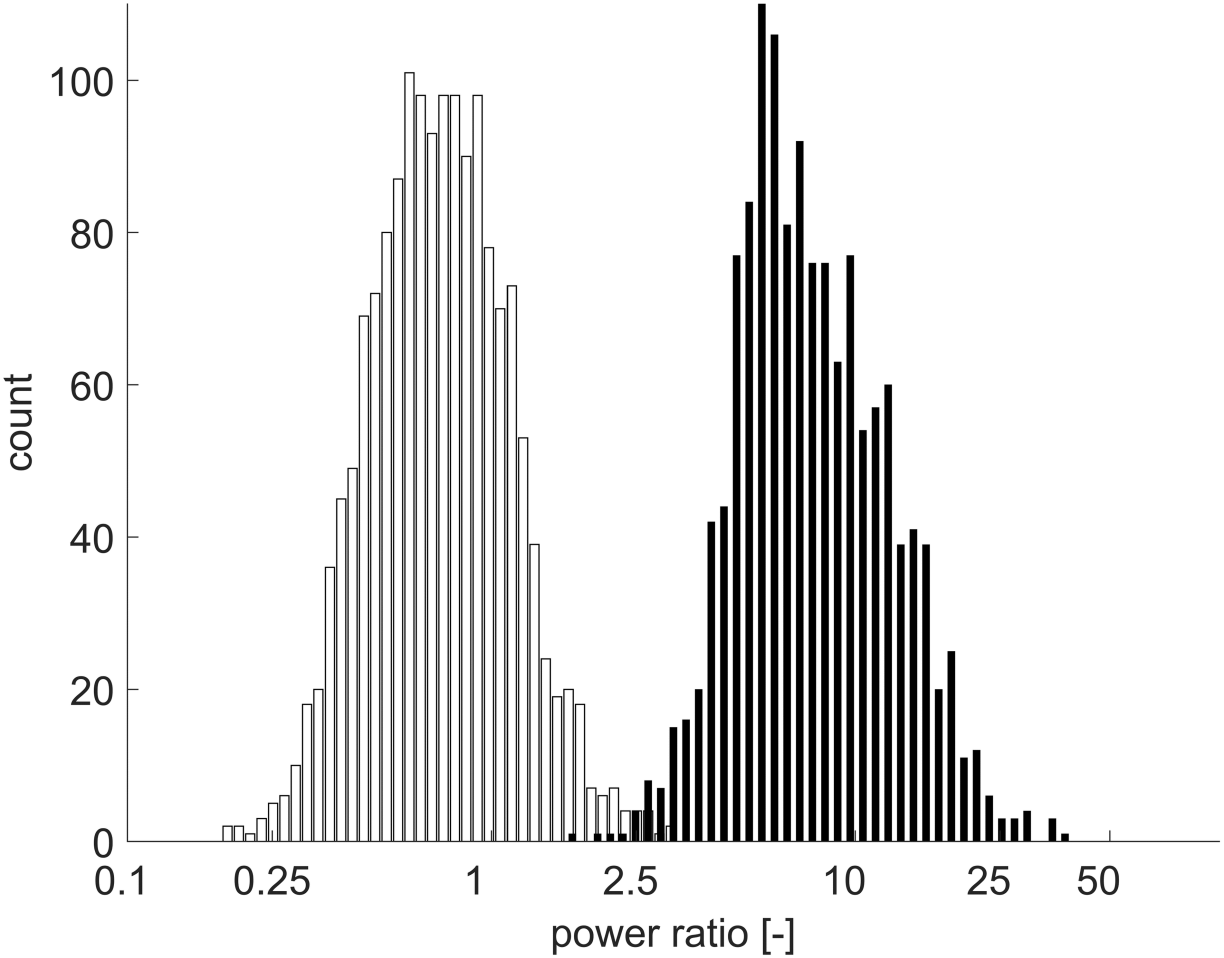
A histogram of power ratios *α* for source components classified as EMG (white) and source components classified as brain signals (black).

To determine the optimal value of the threshold *T*, we computed *α*_*k*,*i*_ for 2992 separated signals ${\hat{s}}_{k,i}\left[n\right]$ that were manually classified as either brain signals or muscular artifacts. The computed *α*_*k*, *i*_ were then used to plot histograms shown in Fig 3, where a clear separation can be seen between the values of *α*_*k*, *i*_ for the individual classes. From Fig 3 we can read that the optimal value of *T* is about 2.5. Thus, a signal ${\hat{s}}_{k,i}\left[n\right]$ was classified as a muscular artifact if *α*_*k*,*i*_ < 2.5.

### Evaluation of algorithm performance

To evaluate the properties of the suggested method, we may apply it to EEG records containing EMG artifacts. This approach has however one serious drawback. While it is possible to observe that the artifacts are suppressed, it is not possible to judge how well the brain signals are reconstructed—with a common EEG record we have no way of knowing what exactly should the EEG look like, when the artifacts are removed. Therefore, this method cannot be objectively evaluated on real world EEG signals corrupted by EMG artifacts.

To circumvent this limitation while keeping the evaluation as realistic as possible, we have devised a testing procedure that uses real world EEG and EMG records, but simulates their mixing using a realistic head model. Specifically, we used the following approach. We started with artifact free EEG signals, henceforth denoted as **x**_*clean*_[*n*]. These signals were recorded on 20 healthy subjects (10 male, 10 female) with age ranging from 19 to 37 years (25.4 ± 5.1 years). During the acquisition of EEG data, the participants were seated with their head supported by a headrest, and instructed to relax. From each subject we obtained a 10-minutes long record with their eyes open and 2-minute long records with their eyes closed. These measurements were approved by a local ethical committee and an informed consent in the written form was obtained from all participants. The data from 111 electrode system with a reference placed on a forehead were digitized with 16 bit resolution and sampled at the frequency of 1024Hz. The data were filtered by a notch filter removing any possible power noise interference at 50Hz and higher harmonics, and by a high pass filter with 1Hz cut-off frequency to suppress the baseline wonder. The channels showing poor electrode connection were visually identified and rejected from further processing. After recording, the EEG was visually checked for artifacts. Special attention was paid to the fringe electrodes that are typically most affected by muscular activity. Only the parts of EEG records with no visually recognizable artifacts were used for testing.

Once, the artifact free EEG signals **x**_*clean*_[*n*] were gathered, we added the muscular artifacts obtained by a simulation on boundary element method (BEM) based realistic head model with the same electrode arrangement as the one used for the EEG measurement. We used a BEM model composed of 19440 elements arranged in 3 layers representing air-skin, skin-skull and skull-brain boundaries. This model was previously used in [24, 25] (see Fig 1 in [25]). In this simulation we placed 5 sources representing cervical muscles and 6 sources representing mandibles into the head model. Temporal signals for these sources were obtained independently for each source from separate surface EMG records measured by electrodes placed on mandibles and across neck during jaws contraction and head movements. Using this combination of EMG signals and realistic head modeling, we created EMG artifacts that could be realistically observed in EEG electrodes. Once the EMG artifacts, henceforth denoted as **x**_*artifact*_[*n*], were generated, they were added to the artifact-free EEG, creating a mixture **x**[*n*] that was subsequently processed by the newly suggested and other state-of-the-art algorithms

$$x\left[n\right]=x_{clean}\left[n\right]+\sqrt{\eta }\cdot x_{artifact}\left[n\right].$$ (20)

The constant *η* was chosen as

$$\eta =\frac{\sum_{n=1}^{N}{∥x_{clean}\left[n\right]∥}^{2}}{\sum_{n=1}^{N}{∥x_{artifact}\left[n\right]∥}^{2}}\xi ,$$ (21)

where ∥.∥ is the Euclidean vector norm, and the *ξ* sets the ratio between the energy of signals **x**_*clean*_[*n*] and the energy of signals **x**_*artifact*_[*n*]. For testing we choose *ξ* = 0, *ξ* = 1 and *ξ* = 4 (the first setting provides artifact free EEG, the second setting makes the energy equal, and the last setting makes the artifact energy four times stronger).

**Fig 4:**
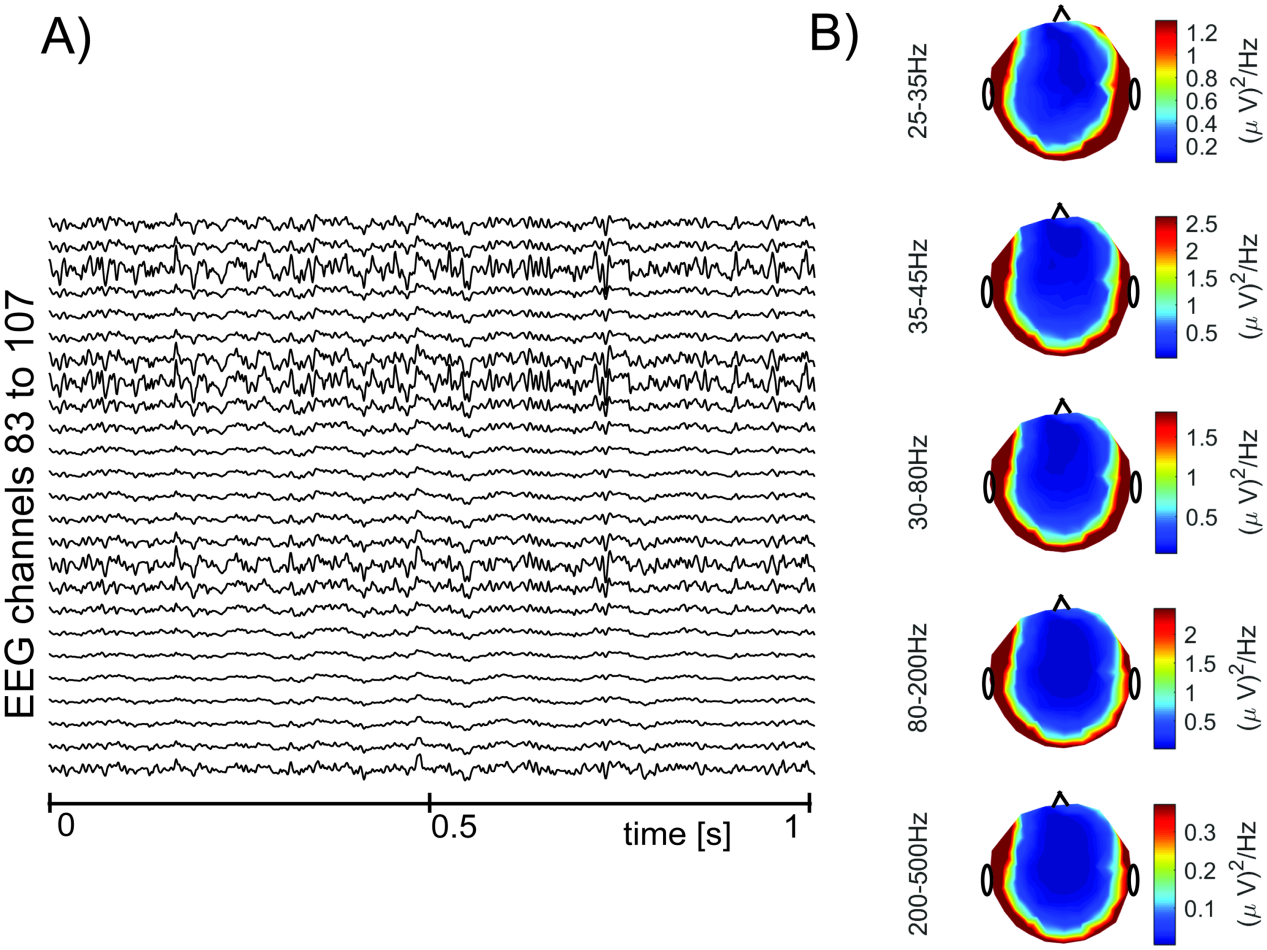
An example of EEG with the simulated EMG contamination. (A) 25 EEG channels with simulated EMG contamination. (B) Topographic maps of average power spectral density in various frequency bands.

**Fig 5:**
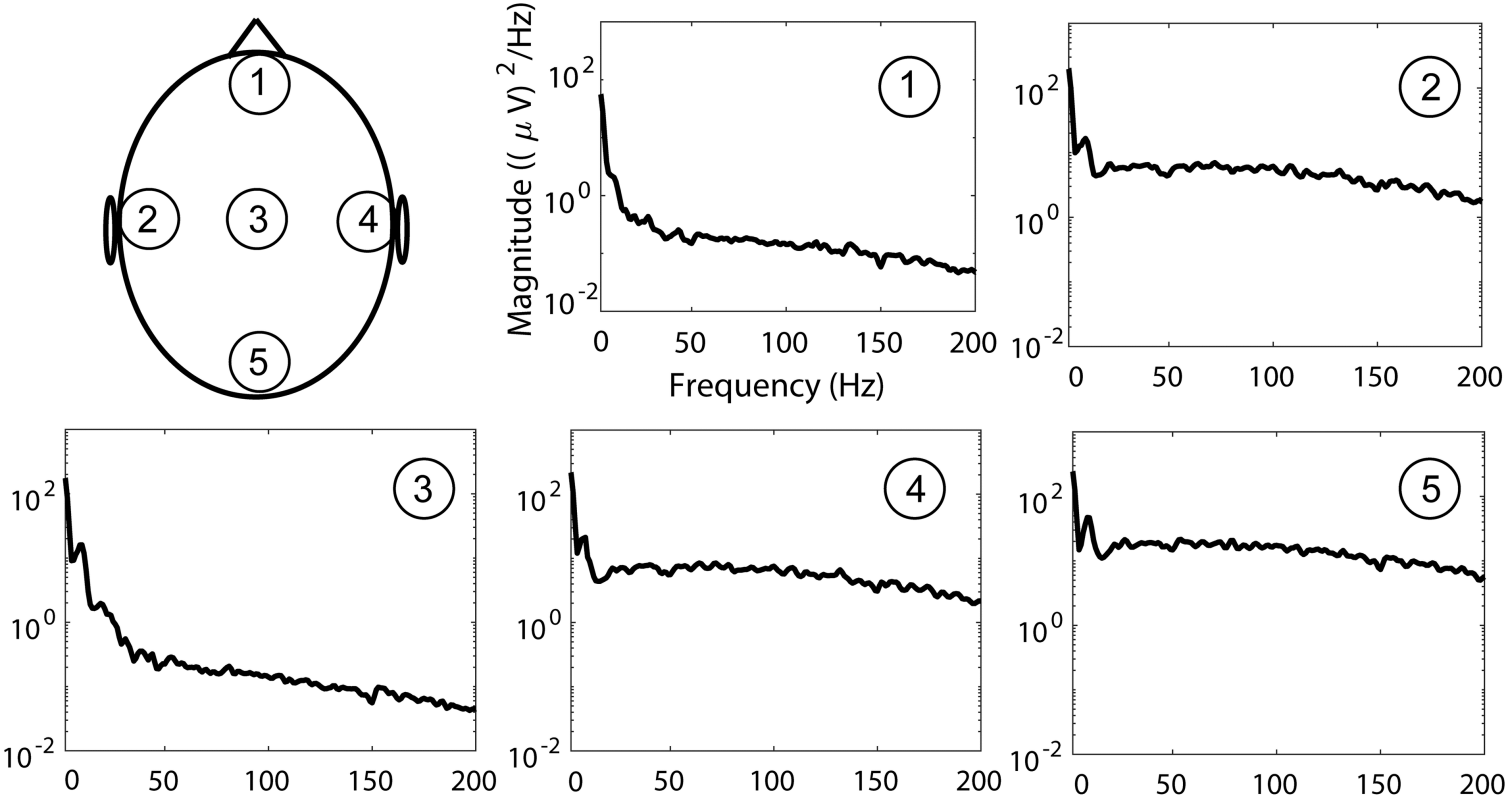
Examples of PSDs of EEG with simulated EMG contamination at several scalp locations.

Illustrations of signals **x**[*n*] obtained through this approach for *ξ* = 1 are in Figs 4 and 5. Fig 4 shows signals in temporal domain, and presents topographic maps of average power spectral density (PSD) in various frequency bands. Fig 5 shows the PSDs of EEG with simulated EMG contamination at five different scalp locations. PSDs in Fig 5 were estimated by averaging periodograms computed from 700 segments, which were 1s long and were weighted by a Hamming window.

Once the reconstructed signals $\tilde{x}\left[n\right]$ were obtained, the quality of their reconstruction was evaluated using an average correlation coefficient

$$r=\frac{1}{M}\sum_{m=1}^{M}\frac{\sum_{n=1}^{N}{\tilde{x}}_{m}\left[n\right]x_{clean,m}\left[n\right]}{\sqrt{\sum_{n=1}^{N}{\tilde{x}}_{m}^{2}\left[n\right]\sum_{n=1}^{N}x_{clean,m}^{2}\left[n\right]}}.$$ (22)

Because the values of *r* are random numbers, the comparison between the values of *r* obtained for various methods necessitates the use of statistical testing. We therefore obtained signals **x**[*n*] 35000 times, applied the above-mentioned methods to each of these trials, averaged the resulting values of *r* obtained for each trial into one mean value $\bar{r}$, and computed their standard deviation *σ*. These values were then compared with the one way ANOVA and the post-hoc Scheffe test.

The evaluation described above was used for two purposes. First, we examined the performance of the newly suggested method for various values of *L* and three different electrode systems (111 electrode system, 10-10 electrode system and 10-20 electrode system). The results were used to identify the values of *L*, for which the newly suggested method provides the best results. Second, we examined and compared the performance of all the above mentioned methods (the newly proposed method and the state-of-the-art methods)—for each method we computed the value of $\bar{r}$, and used these values to compare how well the individual methods remove EMG artifacts.

The evaluation is performed for EEG records that are 1s long (in practice a longer EEG record would be segmented to these shorter segments, and each segment would then be processed separately by an artifact removing algorithm). We find this segment length sufficient for the artifact removal, but short enough to accommodate the nonstationarity of EMG artifacts.

Last, to further test whether the proposed algorithm does not corrupt useful information in EEG data, we followed approach from [11], and checked whether our algorithm does not impair the detection of changes in alpha activity occurring when the measured subjects open or close their eyes. For this purpose, we used two minute long EEG records measured with subjects’ eyes closed, and two minute long EEG records measured with subjects’ eyes open. We used only records from the subjects that manifested distinct augmentation of alpha activity when their eyes were closed (based on visual inspection of signals in the time domain). This was the case in 13 out of 20 participants. The measured signals were processed by the proposed method using segmentation into 1 second long segments. To examine the effect of our method, we estimated PSDs before and after its application. The PSDs were computed for each subject using signals from an electrode that would correspond to the electrode OZ of the 10-10 electrode system. The PSDs estimation procedure was based on Welch’s method with segmentation into 1s long segments with 75% overlap and weighting with the Hamming window. Last, the individual PSDs were averaged over subjects providing the average PSDs presented in section Results.

## Results

**Fig 6:**
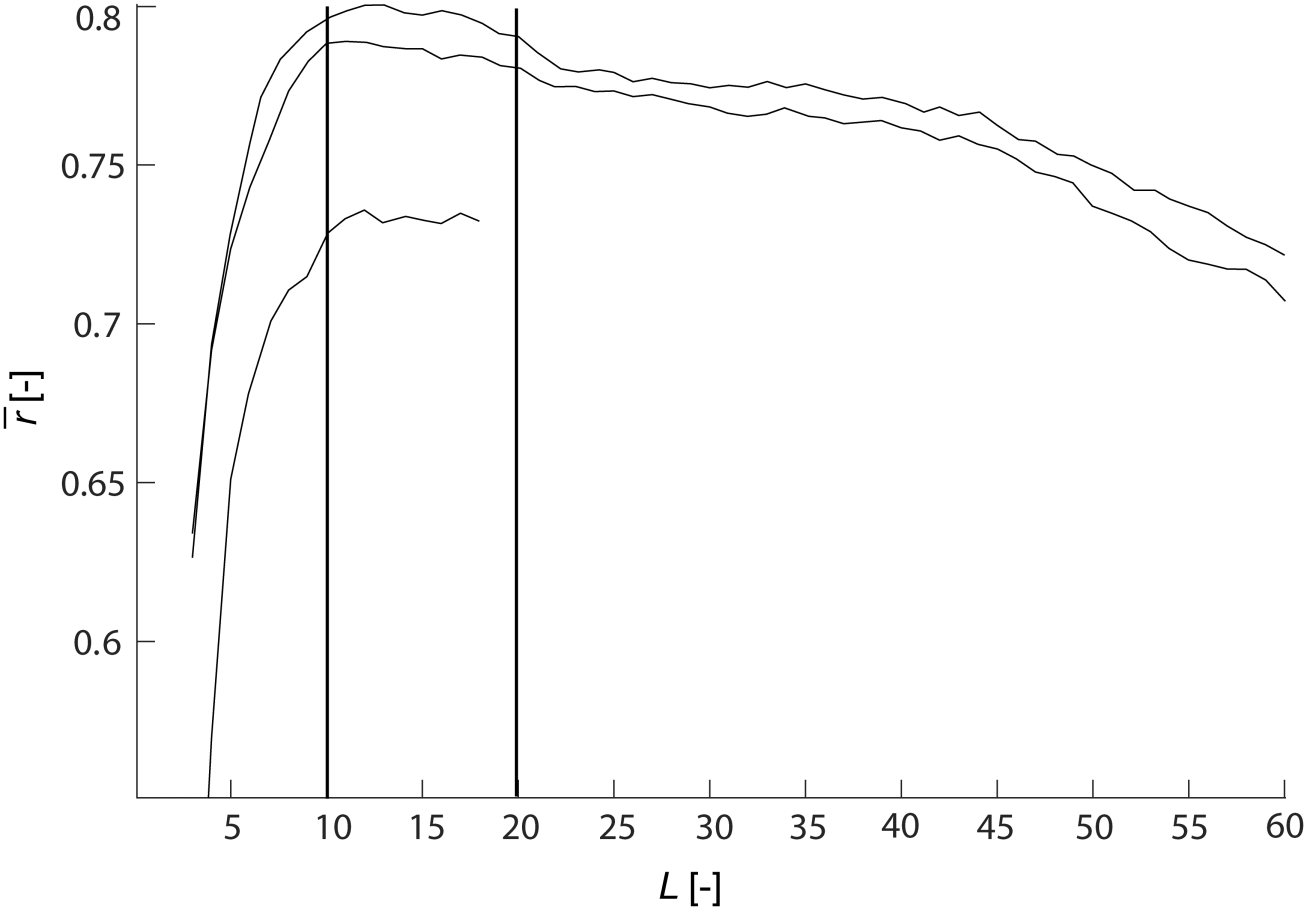
Mean correlation coefficients $\bar{r}$ between the original clean EEG signals and EEG signals with suppressed muscular artifacts as a function of subspace dimension *L* for 111, 10/10 and 10/20 electrode systems.

The Fig 6 shows the values of $\bar{r}$ computed with different subspace dimensions *L* for various electrode systems (*ξ* was chosen as 4). Note that for all electrode systems the best performance is achieved with *L* within interval 10-20. For further computations we chose value *L* = 12, and we suggest to use this value with future applications of our algorithm (the optimization of *L* does not need to be repeated).

We have also performed this optimization for the signal length of 2s and 4s, and found very similar results: the best performance was achievable for *L* within interval 10-20.

Tables 1, 2 and 3 show the values of $\bar{r}$ and *σ* obtained for the suggested algorithm (with *L* = 12) and other state-of-the-art algorithms. The last column indicates whether a statistically significant difference (SSD) was found between the sets of values *r* for respective method and the newly proposed method. These results were obtained for a reference electrode placed on a forehead. We also computed these values with a common average reference and also with data re-referenced to several randomly picked electrodes and always achieved virtually identical results.

**Table 1:** The correlation coefficients from the processing of EEG signals without muscular artifacts (*ξ* = 0).

Method	$\bar{r}$	*σ*	SSD
None	1.0000	0.0000	no
C, 50 PCs	0.8901	0.0536	yes
C, 25 PCs	0.9209	0.0654	yes
C, 12 PCs	0.9689*	0.0465	no
D, 7 PCs	0.2295	0.0857	yes
D, 3 PCs	0.3502	0.0852	yes
D, 2 PCs	0.4053*	0.0891	yes
A	0.8495	0.0483	yes
Ba	0.8601	0.0542	yes
Bb	0.8805	0.0484	yes
Proposed method	0.9831	0.0236	-

Symbol * denotes the best achieved performance in the case of methods C and D.

**Table 2:** The correlation coefficients from the processing of signals with equally strong muscular artifacts and EEG (*ξ* = 1).

Method	$\bar{r}$	*σ*	SSD
None	0.8738	0.0513	yes
C, 50 PCs	0.8182	0.0548	yes
C, 25 PCs	0.8253	0.0627	yes
C, 12 PCs	0.8803*	0.0536	yes
D, 7 PCs	0.2169	0.0691	yes
D, 3 PCs	0.3309	0.0832	yes
D, 2 PCs	0.3976*	0.1040	yes
A	0.7972	0.0569	yes
Ba	0.7909	0.0657	yes
Bb	0.8639	0.0328	yes
Proposed method	0.9536	0.0381	-

Symbol * denotes the best achieved performance in the case of methods C and D.

**Table 3:** The correlation coefficients from the processing of signals with muscular artifacts four times stronger than EEG (*ξ* = 4).

Method	$\bar{r}$	*σ*	SSD
None	0.5631	0.0834	yes
C, 50 PCs	0.5408	0.0951	yes
C, 25 PCs	0.5684	0.1201	yes
C, 12 PCs	0.7061*	0.1057	yes
D, 7 PCs	0.2390	0.0912	yes
D, 3 PCs	0.3831	0.1109	yes
D, 2 PCs	0.4049*	0.0734	yes
A	0.5251	0.0641	yes
Ba	0.5237	0.0561	yes
Bb	0.5879	0.0328	yes
Proposed method	0.8053	0.0694	-

Symbol * denotes the best achieved performance in the case of methods C and D.

The individual state-of-the-art methods are distinguished by the following letters:

A….a plain FastICA [17, Eq (14, 21, 24)] used on data that were pre-whitened by PCA [22] with no overlearning suppression,
Ba…the high pass filtering method, using a fixed high pass filter with 1Hz cut off frequency [13, 14],
Bb…the high pass filtering method, using the AR model based high pass filtering [13, 14],
C….the PCA based method [13, 14],
D….the combination of the PCA and linear regression [3].

To present the best performance for the state-of-the-art methods we show the results for methods C and D with different numbers of principal components and the best performing number of principal component (this number was found by the evaluation of all possible numbers of principal components, and cannot be realistically achieved in practice, where we have no way of knowing which result provides the highest $\bar{r}$).

To assess the dimensionality reduction caused by the newly proposed method, for each D we computed how many strongest singular values comprise 99% of the energy of all singular values. On average, we found this to be 78 for *ξ* = 0, 67 for *ξ* = 1 and 54 for *ξ* = 4. Note that these numbers are much higher than the number of PCs retained in the application of method C (Tables 1, 2 and 3). Therefore, our approach causes much lower dimensionality reduction, and consequently smaller corruption of clean EEG data.

In addition to the quantitative analysis provided above, we also present some illustrative results achieved when our method was applied to real EEG signals.

**Fig 7:**
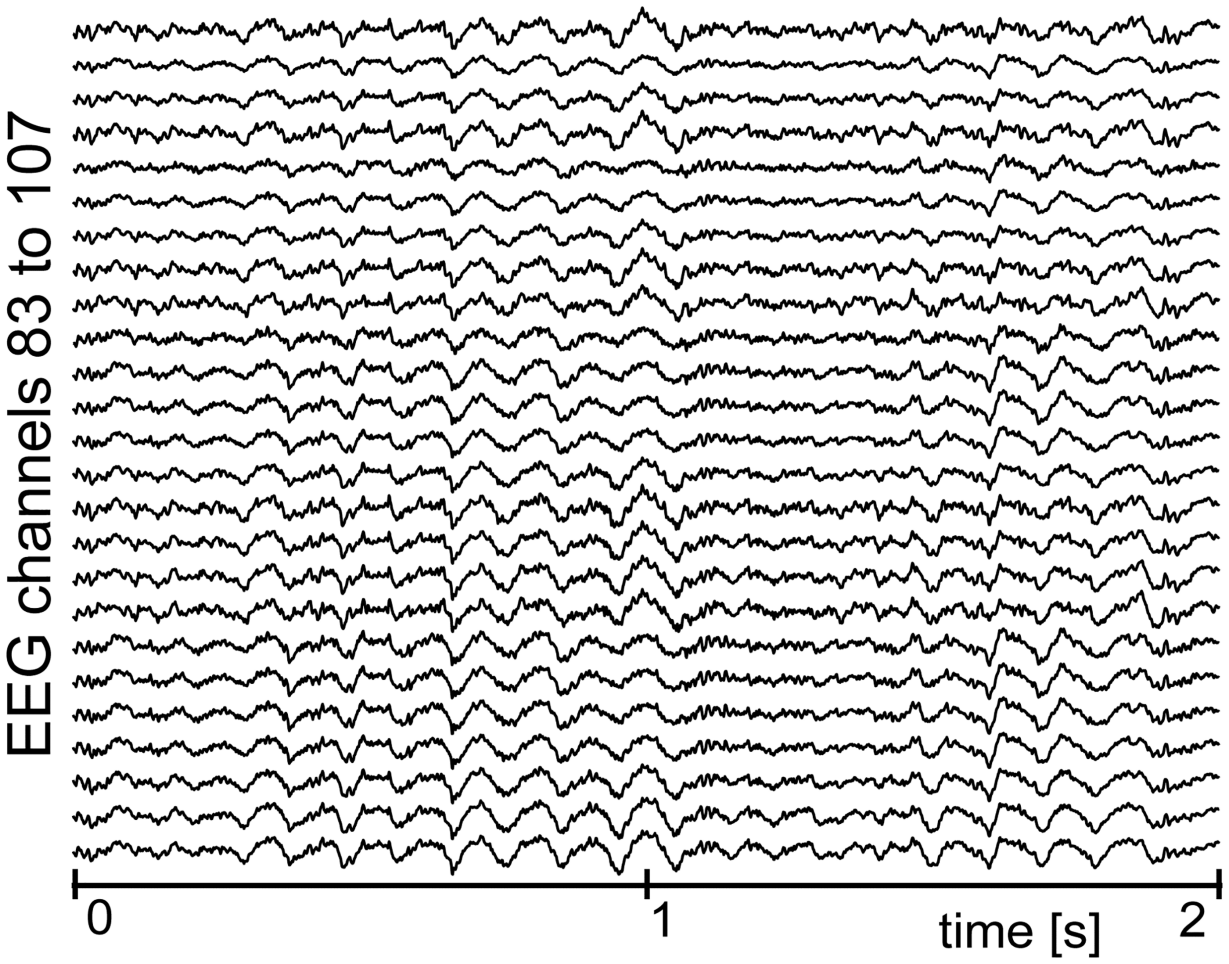
An example of EEG signals processed by the newly proposed method. The original signals are shown in Fig 2A. The EEG contains 111 channels, but for spatial reasons we show only 25 channels that correspond to the ones shown in Fig 2A.

**Fig 8:**
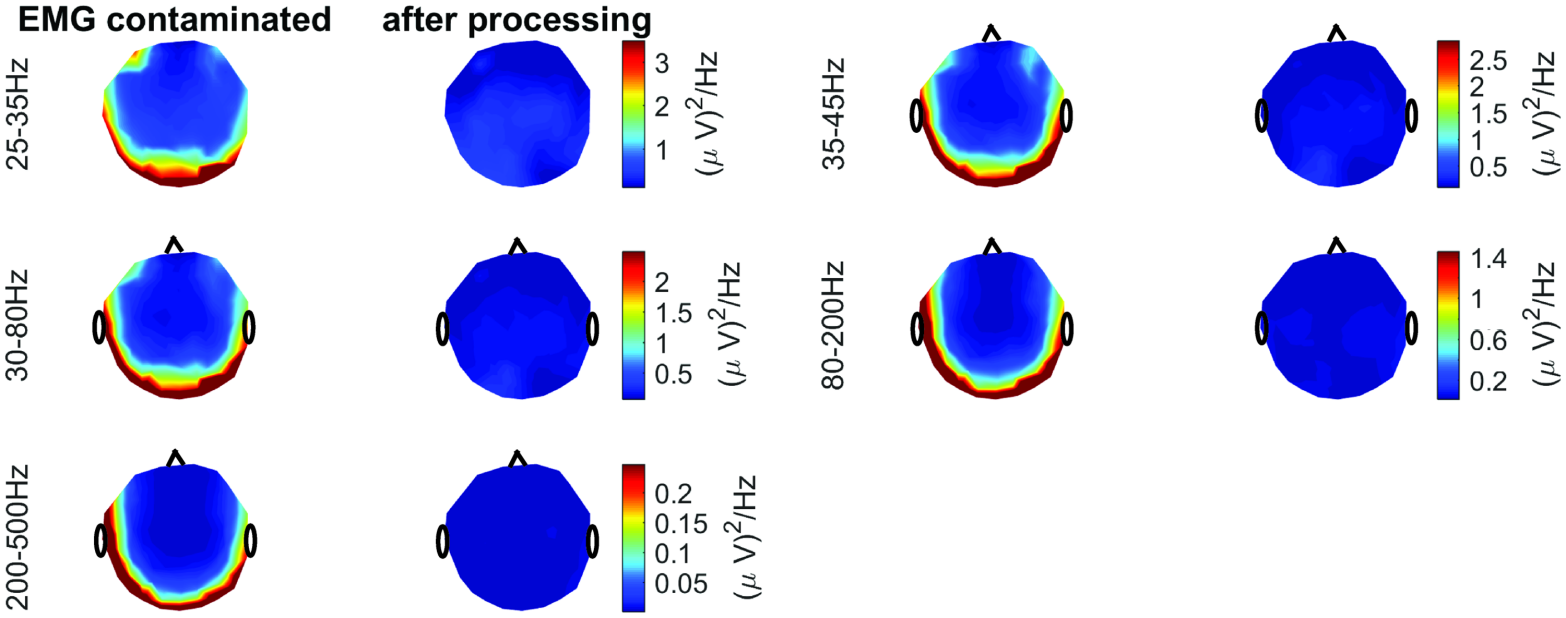
Topographic maps of the average power spectral density in various frequency bands before and after processing by the proposed algorithm.

**Fig 9:**
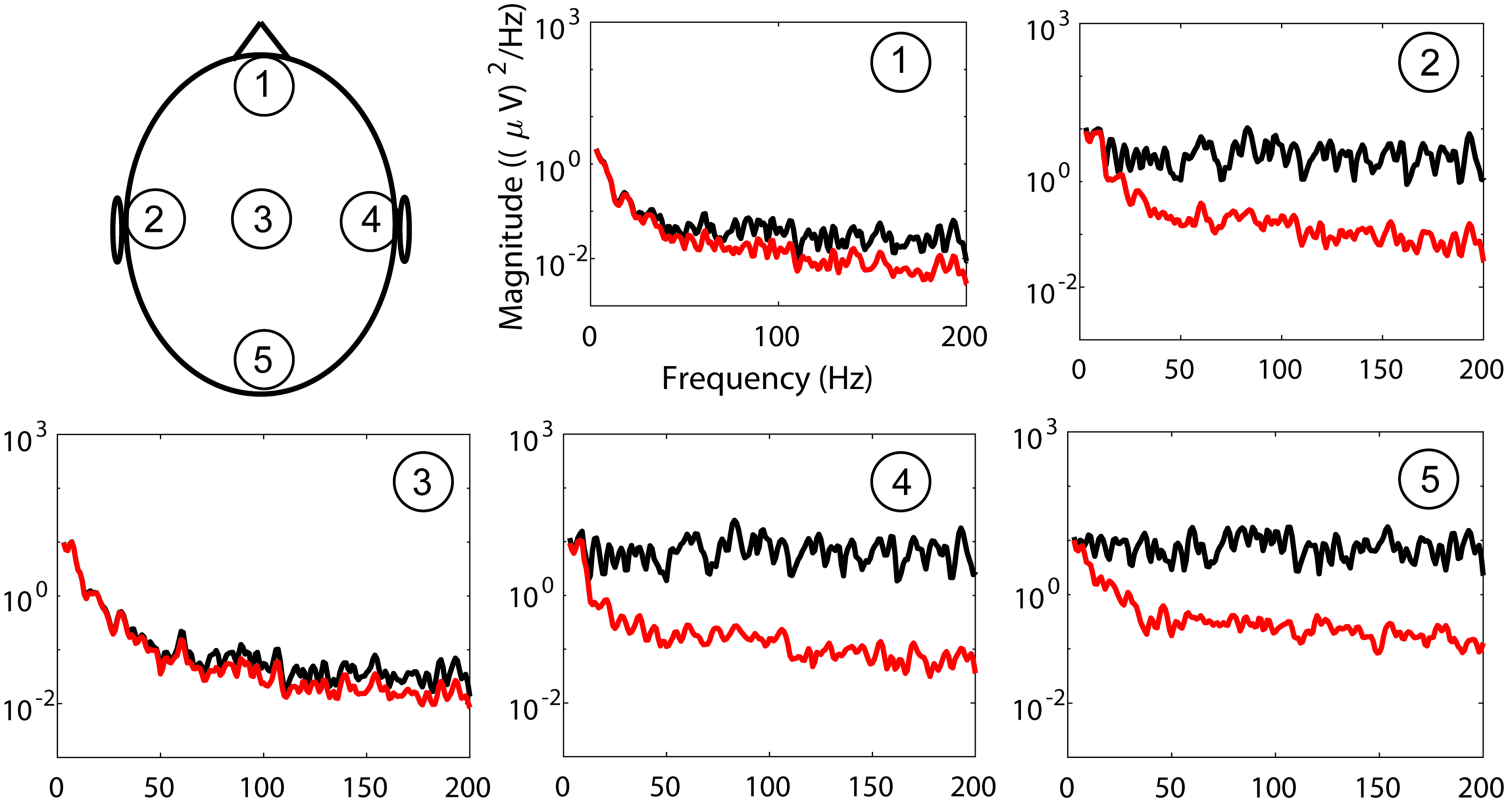
Power spectral densities at several scalp locations before and after processing by the proposed algorithm.

In Fig 7 we show the results obtained when the proposed method was applied to the real EEG signals with EMG artifacts shown in Fig 2A (the signal was split to 1s long segments and our method was applied separately in each of these segments). Fig 7 shows that the EMG artifacts were nicely removed, while the effects of overlearning (shown in Fig 2B) were not observed in any of the estimated source subsets ${\;\hat{\;S}}_{k}\left[n\right]$ during the processing. In addition, Fig 8 shows the topographical power distribution of these signals before and after the application of our method. In Fig 9 we also show the PSDs computed for five different positions on the scalp (these PSDs were computed using Welch’s method with 0.5s long segments overlapped by 75% and weighted by the Hamming window). Note the decrease of power in fringe electrodes, which is associated with the removal of muscular artifacts. These results are similar to those previously presented in [11].

Overall, the results shown in Figs 7, 8 and 9 show nice suppression of EMG artifacts without any noticeable effects of overlearning. However, as stated above, this kind of application cannot be used to quantitatively evaluate the quality of EEG reconstruction; therefore, we include it for illustrative purposes only.

**Fig 10:**
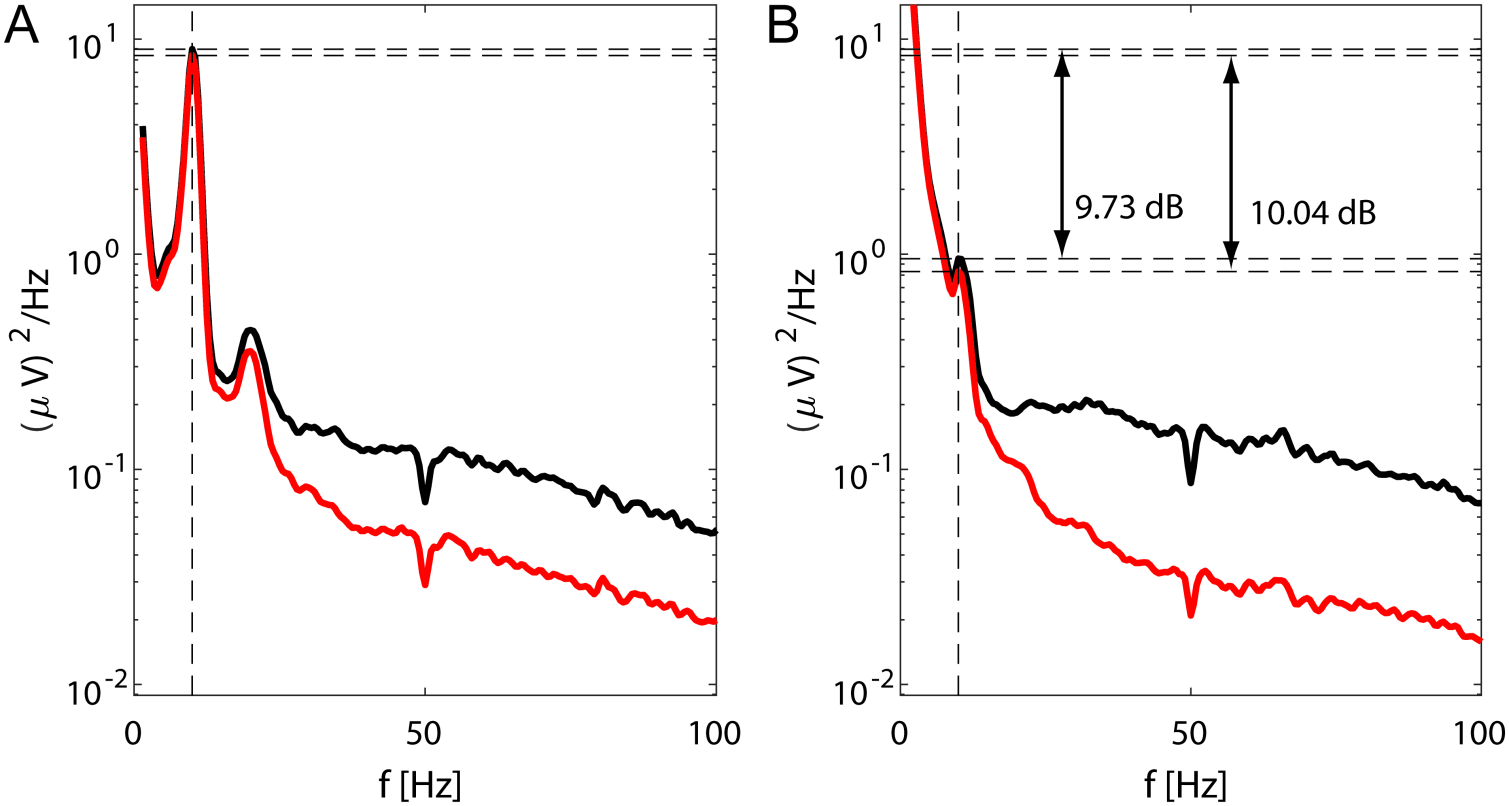
Average PSDs showing changes in alpha power band for eyes open and closed. (A) Average PSDs for resting EEG measured with closed eyes. (B) Average PSDs for resting EEG measured with open eyes. The black lines are PSDs computed from the unprocessed data, while the red ones are PSDs computed from the data processed by the proposed algorithm.

Last, Fig 10 illustrates the changes in the alpha band power in EEG measured on subjects with eyes open and closed. The average PSDs clearly indicate the increase of alpha band power when subjects’ eyes were closed. More importantly, Fig 10 also shows that this change does not seem to be impaired by the application of our method.

## Discussion

The results show that the newly proposed method outperforms all the presented state-of-the-art methods.

The presented results also confirm the weaknesses of the individual state-of-the-art methods pointed out in our introduction. The PCA based dimensionality reduction (method C) distorts the EEG even if no artifacts are being removed. This can be seen as decrease of $\bar{r}$ for clean EEG (*ξ* = 0). The high pass filtering methods (methods Ba and Bb) show consistently poor performance for all setups, because with their use the overlearning is not suppressed. Method D performs poorly for artifact free EEG (*ξ* = 0), where the strongest principal components are composed of brain signals only, and are incorrectly removed from the original EEG. For the stronger artifacts the performance improves; however, it is never as good as for the newly proposed method.

Further, we would like to discuss some additional points related to our method.

In this paper we concentrate primarily on EMG artifacts caused by transient muscular contractions (henceforth, transient EMG artifacts). Besides these artifacts, EEG can also be contaminated by EMG artifacts caused by tonic muscular contraction (henceforth, tonic EMG artifacts). The tonic EMG artifacts can be quite weak, even difficult to recognize upon visual inspection, and of little concern in some applications (e.g. see a small effect of tonic EMG artifacts on evoked response potentials in [11]).

We of course expect that our method will suppress even the tonic artifacts to some extent—compared to transient EMG artifacts they are just weaker and more stationary. Therefore, as long as the ICA is able to separate them, they will be identified and removed from the EEG. In fact, the decrease of power on higher frequencies shown in Fig 10 can likely be attributed to the suppression of tonic EMG artifacts, and this suppression is similar to the results presented in [11].

However, in principle, a stationary interfering source will be better removed using a longer EEG record. Therefore, if a researcher faces a situation where any residual tonic EMG artifacts remaining after the application of our method are still concerning, the application of our method can be followed by the application of a ‘classical’ EMG removal procedure (e.g. [11]) that operates on longer EEG records. In this arrangement our method would be better suited to deal with transient EMG artifacts, and the ‘classical’ EMG removal procedure would be better suited to remove any residual tonic EMG artifacts. In applications where the presence of residual tonic EMG artifacts is not concerning, the application of our method alone would be sufficient.

In relation to tonic EMG artifacts, one could additionally point out that the EEG records *x*_*clean*_[*n*] may not be completely devoid of EMG artifacts, because the tonic EMG artifacts are always present, unless the measurement is performed in paralysis [11, 23]. However, we would argue that while their presence cannot be excluded, visually unrecognizable tonic EMG artifacts have total power much smaller than the total power of EEG, and therefore the effect of these tonic EMG artifacts on the average correlation coefficients $\bar{r}$ will be negligible, especially when we work with transient EMG artifacts with energy equal or greater than the energy of EEG.

Another point that we would like to address is related to our EMG classifier. Note that the classifier described in section Artifact classification is not the only possible approach. For example, work [11] suggested a classifier based on the slope of a power spectrum. In fact, we have also retested our method with the classifier from [11] and obtained virtually identical results. The reader should note that the classifier itself is not an unalterable part of the proposed method, and if necessary a researcher can adjust it to suit application-specific needs.

Further, in the motivation and evaluation of our method, we have limited ourselves to the removal of EMG artifacts, while making few comments about other types of artifacts. This was mostly dictated by a need to fit our presentation into a single paper, and it does not mean that the presented method cannot be generalized for other type of artifacts. In fact, as long as the ICA is able to extract the artifacts into separate components (which was already demonstrated for many different types of artifacts [3, 4, 16]), and we are able to classify these components respectively, the algorithm will allow to remove these components without the manifestation of overlearning.

In this paper we limit ourselves to EMG artifacts only, but because EMG artifacts are an omnipresent nuisance in a great number of EEG records, to have an algorithm that removes these artifacts alone, is, in our opinion, a quite useful contribution.

Regarding the future work, there are also other blind source separation algorithms that might be used for the suppression of EMG artifacts (e.g. SOBI [26], SSA [27], CCA [28]). It may be interesting to explore their overlearning properties, and compare their performance with the ICA based ones. However, before doing so we find it important to first improve the ICA based algorithm, as we attempt to do in this paper.

## Conclusion

This paper presents an ICA based algorithm for the removal of muscular artifacts that is not prone to overlearning for short signals. This allows its application to short signal segments, which makes it better suited for the removal of artifacts that are distinctly non-stationary and appear, disappear or change their origin within a time frame of a few seconds or less. Thus, the algorithm can remove the artifacts present only in short segments to which it is applied. If the artifacts are not detected, the original EEG is not distorted. If the composition of artifacts is changed, the algorithm can react quickly.

We show that the proposed algorithm outperforms all the current state-of-the-art approaches, and provides the best reconstruction of the original EEG.

The algorithm was presented for the purpose of muscular artifact removal; however, we note that it can be easily extended for the removal of other types of artifacts.

Because the muscular artifacts are a recurring problem in a great number of EEG records, we believe that the presented algorithm will provide a useful and efficient tool for their removal.
